# EEG functional connectivity in infants at elevated familial likelihood for autism spectrum disorder

**DOI:** 10.1186/s13229-023-00570-5

**Published:** 2023-10-07

**Authors:** Christian O’Reilly, Scott Huberty, Stefon van Noordt, James Desjardins, Nicky Wright, Julie Scorah, Sara Jane Webb, Mayada Elsabbagh, BASIS team, Simon Baron-Cohen, Patrick Bolton, Susie Chandler, Tony Charman, Janice Fernandes, Holly Garwood, Kristelle Hudryx, Mark H. Johnson, Leslie Tucker, Agnes Volein

**Affiliations:** 1 Department of Computer Science and Engineering, University of South Carolina, Columbia, SC USA; 2 Artificial Intelligence Institute of South Carolina, University of South Carolina, 1112 Greene St, Columbia, SC 29208 USA; 3 Carolina Autism and Neurodevelopment Research Center, University of South Carolina, Columbia, SC USA; 4 Azrieli Centre for Autism Research, Montreal Neurological Institute-Hospital, McGill University, Montreal, Canada; 5 Department of Psychology, Mount Saint Vincent University, Halifax, NS Canada; 6 Compute Ontario, St. Catharines, Canada; 7 Department of Psychology, Manchester Metropolitan University, Manchester, UK; 8 Seattle Children’s Research Institute, Seattle, WA USA

**Keywords:** Autism spectrum disorder, Functional connectivity, Sex differences, Infants, Source reconstruction, Electroencephalography, ADOS, Sibling studies, Longitudinal

## Abstract

**Background:**

Many studies have reported that autism spectrum disorder (ASD) is associated with atypical structural and functional connectivity. However, we know relatively little about the development of these differences in infancy.

**Methods:**

We used a high-density electroencephalogram (EEG) dataset pooled from two independent infant sibling cohorts, to characterize such neurodevelopmental deviations during the first years of life. EEG was recorded at 6 and 12 months of age in infants at typical (*N* = 92) or elevated likelihood for ASD (*N* = 90), determined by the presence of an older sibling with ASD. We computed the functional connectivity between cortical sources of EEG during video watching using the corrected imaginary part of phase-locking values.

**Results:**

Our main analysis found no significant association between functional connectivity and ASD, showing only significant effects for age, sex, age-sex interaction, and site. Given these null results, we performed an exploratory analysis and observed, at 12 months, a negative correlation between functional connectivity and ADOS calibrated severity scores for restrictive and repetitive behaviors (RRB).

**Limitations:**

The small sample of ASD participants inherent to sibling studies limits diagnostic group comparisons. Also, results from our secondary exploratory analysis should be considered only as potential relationships to further explore, given their increased vulnerability to false positives.

**Conclusions:**

These results are inconclusive concerning an association between EEG functional connectivity and ASD in infancy. Exploratory analyses provided preliminary support for a relationship between RRB and functional connectivity specifically, but these preliminary observations need corroboration on larger samples.

**Supplementary Information:**

The online version contains supplementary material available at 10.1186/s13229-023-00570-5.

## Background

Heterogeneity in the causes, symptoms, and impact of autism spectrum disorder (ASD) represents a fundamental challenge to preclinical, clinical, and translational research. While genetic and non-genetic factors contribute to autism susceptibility [1, 2], these factors are also likely to contribute to susceptibility for a broader range of neurodevelopmental disorders. Adding a layer of complexity is the fact that these factors interact over time, modifying early brain development and leading to heterogeneous outcomes in terms of distinct functional, cognitive, and language dimensions not well captured by narrow diagnostic categories [3–6].

The biological sex has emerged as another important contributor to this heterogeneity. The presence of sex-based differences in the prevalence and clinical presentation of autism is well-established [7], and it plays a significant role in the neurobiology of autism [8]. Functional connectivity—a measure of statistical dependencies between the activity in distinct brain regions—is of particular interest because functional networks emerge during infancy when brain plasticity is at its peak and autism symptoms begin to appear [9]. In the general population, higher functional connectivity is observed in females relative to males for whole-brain connectivity and in functionally distinct networks, including the default mode network and the central executive network [10, 11]. Despite some inconsistencies, a systematic literature review suggested moderate support for a general pattern of long-range EEG and magnetoencephalography (MEG) underconnectivity distinguishing autistic from neurotypical individuals [12]. These participant samples have often been heavily skewed toward males, resulting in a gap in understanding the development of functional networks in autism in males relative to females. The few studies that have explored the effect of biological sex on functional connectivity in autism have reported that autistic females show increased connectivity compared to both autistic males [13] and neurotypical females [14], while another study reported reduced functional sensorimotor connectivity associated with ASD symptoms specifically in females [11].

Despite the rapid increase in studies on early trajectories of brain development in infants who later develop autism [9], little is known about early functional connectivity. Only a few ASD studies have estimated connectivity in infancy [15–19]. While the findings have not been conclusive, current evidence suggests that cortical network maturation differs in autistic individuals, with initial overconnectivity within the first year of life followed by underconnectivity beginning in toddlerhood [12]. Few infant-sibling studies have included biological sex as a variable of interest when studying functional connectivity in ASD, with no significant sex-related results [15, 16]. This situation might be due in part to the study of biological sex in sibling studies being complicated by the relatively low number of children who go on to develop autism and the high sex imbalance (mostly male) in the diagnosed subsample.

Besides the insufficient attention given to sex differences, the focus on categorical diagnostic outcomes has also been criticized, with previous studies finding categorical analyses to mask significant heterogeneity in the nature and severity of symptoms for children who develop autism [20]. This issue also extends to children who do not receive an ASD diagnosis but experience problems in other developmental domains like language and attention [21]. Moreover, dimensional analysis provides an opportunity to integrate developmental trajectories in children at elevated likelihood for ASD who experience ASD symptoms or not and gain further insights into resilience processes [9]. It also provides a better framework to distinguish different dimensions. For example, a previous study reported an association between functional connectivity and later severity of restricted and repetitive behaviors (RRB) measured with the Autism Diagnostic Interview-Revised (ADI-R) in one-year-olds [18, 22]. Another study used a support vector regression with functional connectivity and ADOS scores (Toddler module) and reported that alpha band (6–12 Hz) connectivity (phase coherence) across frontal and temporoparietal regions at three months predicts ASD symptoms at 18 months [16].

In the current study, we aim to study functional connectivity in early development to better understand factors that distinguish such connectivity between infants with an elevated likelihood for autism (ELA; assessed by the presence of a sibling with ASD) compared to a control group of infants with a typical likelihood of autism (TLA; no family history of ASD). We further aim to provide a better account of the effect of biological sex. To address the challenges associated with the heterogeneity of this condition, we utilize the International Infant EEG Platform (EEG-IP). EEG-IP addressed the aforementioned challenges by pooling and standardizing EEG recordings from two studies of ELA and TLA infants [23, 24]. Further, besides reporting results for categorical outcomes, we performed dimensional analyses using ADOS severity scores to address previous critics regarding the use of categorical diagnostic outcomes and distinguish potential relationships with social affect and RRB dimensions.

## Methods

### Sample

The sample used for this study was taken from the EEG Integrated Platform (EEG-IP) [24], which includes ELA and TLA infants from two sites: the Seattle Children’s Hospital (Seattle, Washington, USA) and Birkbeck University (London, UK). For both sites, EEG was collected around 6 and 12 months of age. Clinical diagnostic assessments included the Autism Diagnostic Observation Schedule (ADOS) and were confirmed by clinical judgment. ADOS was administered at around 24 months of age for both sites (ELA only) and around 36 months for all participants (ELA and TLA) in London and a subset of participants in Seattle. Both sites administered the ADOS Generic (ADOS-G) [25]. However, we applied revised algorithms [26] to the ADOS-G scores. ADOS calibrated severity scores [27] were calculated for the social affect and RRB subdomains. All the analyses we report in this paper used these calibrated severity scores (CSS) only. Participants with an unknown outcome (*n* = 4) or TLA later diagnosed with ASD (*n* = 3) were excluded from the analysis. Sample and subsample sizes (including sex distribution) are summarized in Table 1.

**Table 1:** Sample size, for participants that provided EEG recording or EEG and ADOS at 6 and/or 12 months (male/female)

Group	Outcome	London (EEG)	London (EEG & ADOS)	Seattle (EEG)	Seattle (EEG & ADOS)	Total Across Sites (EEG)	Total Across Sites (EEG & ADOS)
ELA	ASD	11/5	11/5	4/7	2/3	15/12	13/8
	No ASD	10/25	10/25	22/6	12/4	32/31	22/29
	Unknown	0/0	0/0	0/0	0/0	0/0	0/0
ELA Total		21/30	21/30	26/13	14/7	47/43	35/37
TLA	ASD	0/0	0/0	2/1	0/0	2/1	0/0
	No ASD	21/27	20/27	21/16	12/10	42/43	32/37
	Unknown	0/0	0/0	3/1	0/0	3/1	0/0
TLA Total		21/27	20/27	26/18	12/10	47/45	32/37
Total across groups		42/57	41/57	52/31	26/17	94/88	67/74

*ADOS* autism diagnostic observation schedule; *ASD* autism spectrum disorder; *EEG* electroencephalogram; *ELA* elevated likelihood for ASD; *TLA* typical likelihood for ASD

### EEG acquisition and pre-processing

EEG was collected as infants watched videos presented on a computer monitor while seated on their caregivers’ laps in a dark room. Infants from the Seattle study viewed a series of age-appropriate videos that included brightly colored toys that moved and produced sounds, alternated with an adult female singing nursery rhymes. Each of these video sets was approximately 60 s in duration. Infants from the London sample watched the same videos, with an additional video of an age-appropriate toy being activated by a human hand. These videos were truncated (compared to the version used in Seattle) to a duration of 30–40 s. The number of trials (i.e., watched videos) depended on the infant’s cooperation. Both sites used a 128-channel Hydrocel geodesic sensor net and Electrical Geodesics (Eugene, Oregon) Net Station software. Scalp EEG was recorded at 500 Hz using a vertex reference and re-referenced offline using a robustly interpolated average. The London and the Seattle datasets were notch-filtered at 50 Hz and 60 Hz, respectively, to remove power line contamination.

Semi-automated pre-processing was done with the EEG-IP-L pipeline [23] using an Octave interpreter running on a Compute Canada cluster. Pre-processing involved comprehensive data annotation to identify artifacts and non-stationarity in scalp channels and independent components. This pipeline used ICLabel [28] to provide an initial automated classification of the independent components as being either valid brain activity or capturing some artifacts, such as electromyographic, electrocardiographic, electrooculographic, and power line contamination. Quality control included an expert review of all data annotations and confirmation of artifacts informed by initial classification, topographies, activation time series, dipole fit, and power spectrum. For an expanded description of pre-processing criteria and artifact thresholds, see [23, 24]. At the time of analysis, the raw EEG was (1) high-pass filtered at 1 Hz, (2) notched filtered at the powerline fundaments frequency and its three first harmonics, (3) channels, segments, and independent components flagged by the EEG-IP-L pipeline were dropped, (4) missing channels were interpolated with spherical splines, (5) “EOG channels” (E1, E8, E14, E17, E21, E25, E32, E125, E126, E127, E128) were dropped, and (6) an average reference was applied. This scalp EEG was then epoched into 1-s non-overlapping windows for source reconstruction and calculation of functional connectivity. We used relatively short time windows, as they have shown to be advantageous for estimating functional connectivity [29].

### Source reconstruction

Most EEG connectivity studies in autism have been performed on scalp electrode signals [12], which are known to have various limitations compared to source analyses, such as poorer signal-to-noise ratio, the impossibility of directly relating observations to brain structures, and the confounding effect of volume conduction, reference electrodes, and common sources [30–34]. Although tools for EEG source reconstruction are now widely available, they have been used only in a few autism studies [35, 36]. For infants with ASD or at elevated likelihood for ASD, the lack of age-matched templates has resulted in the use of head templates built from an adult population, such as the Montreal Neurological Institute (MNI) brain [36], which is likely to distort source estimations in ways that are not well-established. For this study, we used a recently developed set of infant structural head templates [37] to perform EEG cortical source reconstruction, investigate functional connectivity in infants, and identify potential ASD risk and resilience factors. To avoid confounding a potential effect of the head template with the recording time points, we used the 12-month template for both time points. As a validation, the same analyses were performed with age-matched templates and resulted in qualitatively similar conclusions. The cortex for these templates has been parcellated using the Desikan-Killiany [38] scheme. Sources were estimated using MNE-Python 0.23 [39], with the eLORETA inverse operator [40], *λ*^2^ = 10^–4^, and with dipoles aligned perpendicular to the cortical mesh. Sources were averaged for every brain region, using the “mean flip” mode from MNE-Python.

### Functional connectivity

The corrected imaginary part of phase-locking value (CIPLV) [41] was computed between every pair of brain regions. We selected this measure because, as opposed to measures like coherence, its reliance on the imaginary part of the phase-locking value makes it insensitive to unlagged synchrony and, therefore, to the confounding impact of volume conduction. Further, CIPLV has been preferred over phase lag index (PLI) because the latter has been shown to have low test–retest reliability [42, 43], a fact that might come as no surprise given the discretization of the phases differences caused by the use of the sign function [44]. Our experience with these different measures also supports the greater reliability of this measure. We illustrate this observation in Additional file 1: Figure S1e where we show using a randomly selected subject how the standard deviation (normalized by mean values) of bootstrapped samples of connectivity estimates for the weighted PLI (wPLI) is larger than for CIPLV, indicating a superior reliability of CIPLV. All CIPLV values were computed using the spectral_connectivity_epochs from the mne_connectivity package v.0.5.0. Further, as functional connectivity estimates are biased depending on the sample size [45] (see also Additional file 1: Figure S1a for the impact of sample size on CIPLV estimates), we ensured that estimates were all computed using the same number of epochs across subjects by bootstrapping the estimates using repeated samples of 20 epochs (see supplementary information for details). Recordings with less than 20 valid 1-s epochs (*n* = 21/325) were rejected from the analyses.

We initially computed functional connectivity for the broadband signals (3–100 Hz), as well as for a few typical frequency bands (in Hz): theta: [3, 6]; alpha [6, 10]; beta [10, 30]; gamma [30, 100]. Given that our preliminary analyses did not indicate a reliable impact of frequency on between-group differences in connectivity, we report only the broadband analyses. Broadband measures were obtained by averaging connectivity across frequencies. For comprehensiveness, we provide in Additional file 1: Figures S4–S6 a visualization of average connectivity per site, sex, age, group, and frequency.

### Resting-state networks

To compare the functional connectivity estimated in this naturalistic video-watching task within the different resting-state networks, we labeled the brain regions as being part of the auditory, default mode, dorsal attention, salience, or visual networks, or none of the above, following a previously published classification [46]. The functional connectivity for each of these networks was computed as an average of the all-to-all connections between the regions that are part of the corresponding networks.

### Statistical analysis

Statistical analyses were run using pandas 1.1.4 for data manipulation, statsmodels 0.12.2 for linear regressions, and seaborn 0.11.0 and matplotlib 3.4.0 for visualization. To improve the normality of the connectivity measures, we transformed them using the following logit equation:

1 $$\log {\text{it}}\left( {{\text{CIPLV}}} \right) = \log \left( {\frac{{{\text{CIPLV}}}}{{1 - {\text{CIPLV}}}}} \right)$$

This transformation changes the support of the connectivity measures from [0, 1] to [− inf, + inf] and helps diminish the asymmetry of the distribution, particularly the heavy right tail we observed in our empirical distributions. We further rejected the EEG recordings in which the functional connectivity was considered a statistical outlier, defined as being either more than 1.5 inter-quartile intervals above the third quartile or below the first quartile (*n* = 11/299; see supplementary information for details). In total, from 325 recordings, 21 were rejected because they had less than 20 valid 1-s epochs (see Additional file 1: Figure S1.b), 5 were rejected because they came from participants with unknown diagnostic status (see Table 1), and 11 were rejected as outliers (see Additional file 1: Figure S2), leaving a total of 288 recordings for analysis. Table 2 lists the number of available recordings after artifact rejection.

**Table 2:** Sample size available after artifact rejection, specified as male/female

	6 months	12 months
*London*		
*TLA*	12/21	20/21
*ELA-noASD*	6/16	9/21
*ELA-ASD*	9/2	10/5
*Seattle*		
*TLA*	24/15	16/14
*ELA-noASD*	20/6	19/5
*ELA-ASD*	2/5	4/6
*Combined*		
*TLA*	36/36	36/35
*ELA-noASD*	26/22	28/26
*ELA-ASD*	11/7	14/11

Recordings were included if they had at least twenty 1 s epochs of clean EEG and their estimated CIPLV was not a statistical outlier. Total number of valid recordings for analysis: 288

*ASD* autism spectrum disorder; *ELA-ASD* elevated likelihood for ASD-diagnosed with ASD; *ELA-noASD* elevated likelihood for ASD-diagnosed with no ASD; *TLA* typical likelihood for ASD

In our main analysis, we used mixed-effect multifactorial linear regressions to test the impact of biological sex, diagnostic groups (TLA, ELA-noASD, ELA-ASD), site, and age on functional connectivity, using the subject as grouping random-effect factor and the following model structure for the fixed effects (i.e., all main effects and two-way interactions):

2 $$\log {\text{it}}\left( {{\text{CIPLV}}} \right)\sim ({\text{sex }} + {\text{ group }} + {\text{ site}} + {\text{ age}})^{2}$$

Then, we looked at correlations between the overall (i.e., as opposed to the social affect and RRB subscales) ADOS CSS and functional connectivity, within the ELA group using:

3 $$\log {\text{it}}\left( {{\text{CIPLV}}} \right) \, \sim \, ({\text{sex }} + {\text{ ADOS }} + {\text{ site }} + {\text{ age}})^{2}$$

Regression (3) using ADOS CSS includes only the ELA subjects (recordings: 112; participants: 70), whereas the regression using the diagnostic group (2) used recordings from both groups (recordings: 288; participants: 176).

## Results

Below, we proceed with a well-sampled regression analysis of the connectivity in the EEG-IP dataset, exploring categorical diagnostic group effects and the effect of ADOS CSS as a dimension. This analysis constitutes the main outcome of this paper, and it resulted in null findings concerning the association between functional connectivity and ASD. Since our main analysis was purposefully limited in its granularity to avoid losing statistical power by stratification or multiple testing, null findings could have been due to loss of specificity in the measurements (e.g., not distinguishing for factors like functional networks, regions, etc.). Thus, we then proceeded to a more exploratory and descriptive analysis to provide tentative relationships that could indicate future research directions. This exploratory analysis is less statistically powered because of stratification and more sensitive to false positives due to its exploratory nature. Thus, the results from this secondary analysis need to be taken as potential research hypotheses to be corroborated in future studies. In this context, we investigated whether a potential relationship between ASD and connectivity in infancy could be affected by multiple factors, including age, biological sex, site, functional networks, and distance between communicating regions. As described below, we observed a tendency for underconnectivity associated with elevated familial likelihood and later ASD diagnosis, a negative correlation between RRB symptoms and functional connectivity, and potential differences in how ADOS CSS subscales correlate with connectivity between male and female ELA infants.

### Main analysis

Both mixed-effect regressions described in models (2) and (3) show a negative effect of age on functional connectivity (2: *β* = − 0.0148, *p* = 0.000018; 3: *β* = − 0.0112, *p* = 0.055). No other factors were significant (*p* > 0.1) for model (2). For model (3), the site was significant (*p* = 0.024), while sex (*p* = 0.055) and the age-sex interaction (*p* = 0.061) were marginally significant. We ran these models on the overall connectivity (i.e., the connectivity averaged across pairs of regions, within each recording). Equivalent regressions but with the connectivity of every connection have also been tested. Although additional factors were significant, the effect of interest (i.e., a main effect of ADOS or diagnostic group) was not significant in these regressions either. These results are provided in supporting documents (see Additional file 1: Tables S1 and S2).

**Fig. 1:**
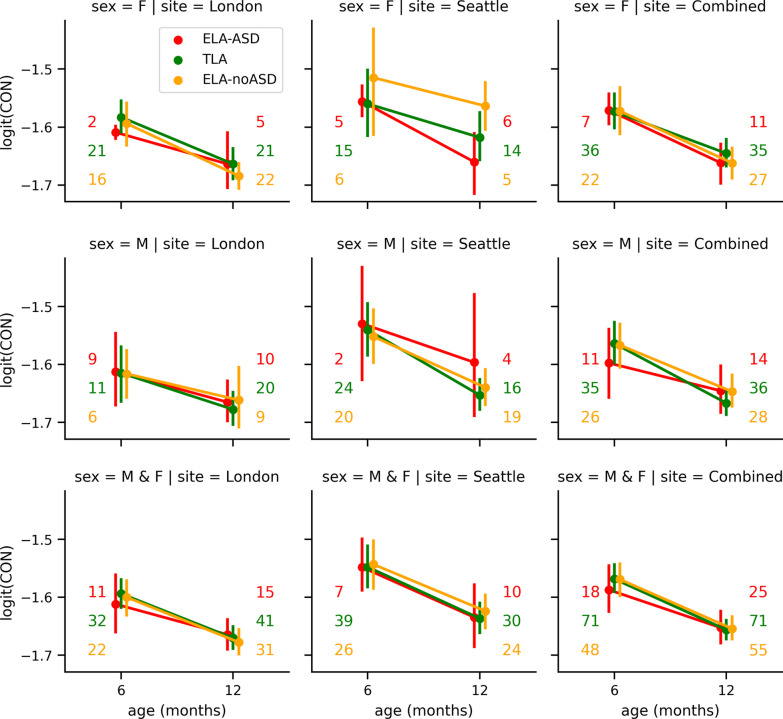
Average logit-transformed CIPLV connectivity. Displayed as a function of the age (*x*-axis), the site (columns), biological sex (row), and the diagnostic outcome groups (color). Whiskers represent the bootstrapped 95% confidence intervals. These plots are for all-to-all connectivity averaged by recording. Displayed are the mean values and their bootstrapped 95% confidence intervals. Numbers next to the whiskers indicate the number of participants for each condition

Figure 1 provides an additional visualization of averages stratified by site, age, sex, and group. This figure confirms the clear decrease in overall connectivity with age, from 6 to 12 months, as statistically assessed by models (2) and (3). In Fig. 1, confidence intervals are obtained with bootstrapping and are for variable sample sizes, as indicated in the figure. Also, as suggested by the linear regression, no other factor than age reliably modulates this effect. However, we would detect only very large effects given the sample sizes. For reference, a two-tail t-test would require 64 participants per group to detect a large effect (Cohen’s *d* = 0.5) at a statistical significance level of *p* < 0.05 and with a power of 0.8. Nevertheless, these results support that such differences in connectivity, if present, are unlikely to be large enough to directly serve as a reliable biomarker for diagnostic purposes in single individuals.

### Exploratory analysis: regional specificity

**Fig. 2:**
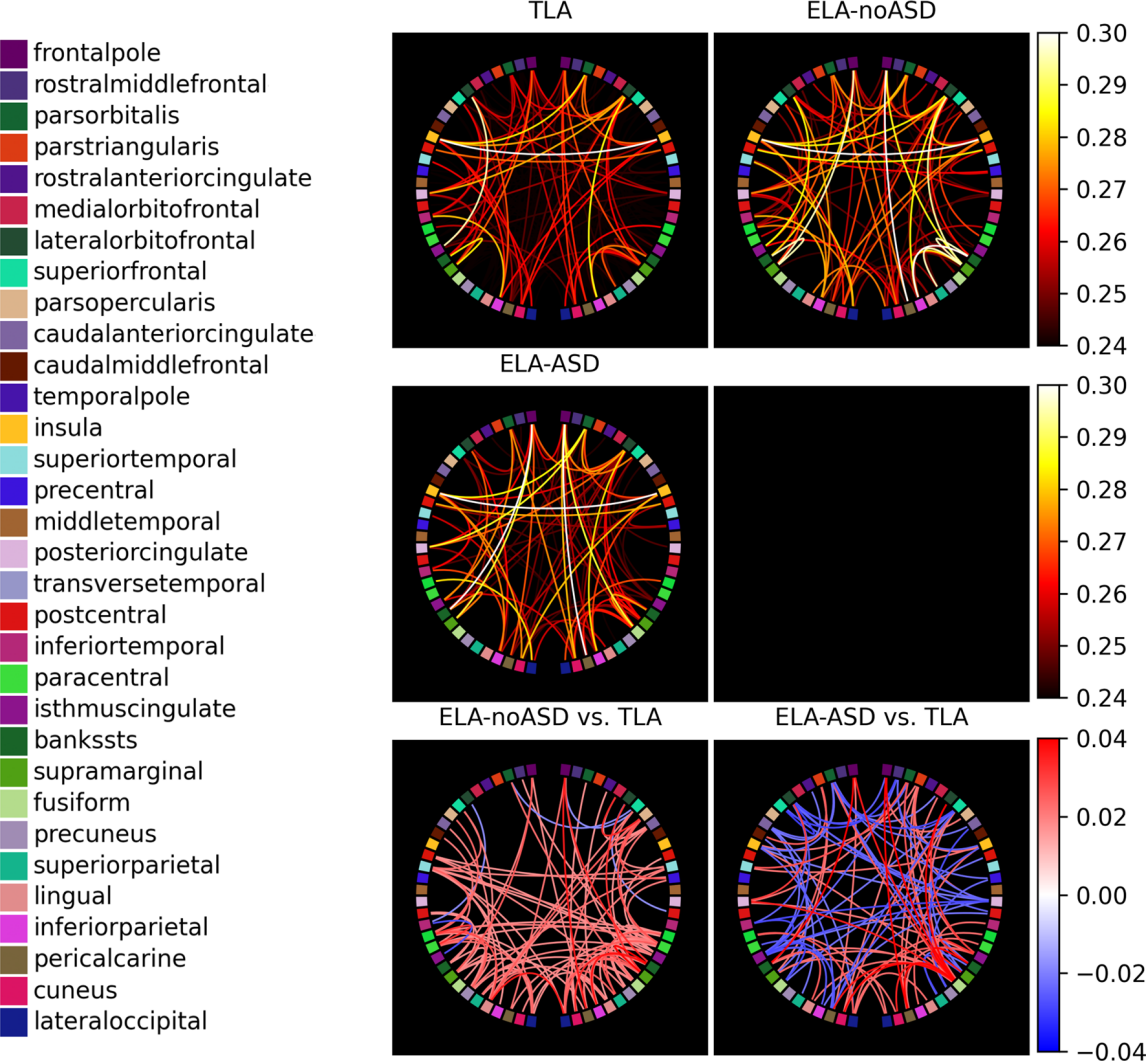
Functional connectivity within and between groups. The three upper circular connectivity plots show the most strongly connected pairs of regions for each diagnostic group. Similarly, the two lower graphs show the strongest differences in connectivity between the TLA and the two subgroups of ELA infants. The three top and two bottom graphs have been plotted using the same color scale to allow fair comparisons. The left (right) strip of circular plots corresponds to the regions of the left (right) hemisphere. Brain regions are color-coded, and their order in the circular plot is the same for each hemisphere (i.e., the superiortemporal region is represented for both hemispheres with the same cyan color, and the regions from the two hemispheres are arranged symmetrically). The position of the regions, from posterior (bottom) to anterior (top), and their color-coding are shown in the legend on the left side of the figure. These plots only show the 100 region pairs with the largest CIPLV connectivity (top three panels) and the 100 pairs with the largest between-group differences in CIPLV connectivity (bottom two panels)

To explore relationships that could have been missed by aggregating functional connectivity, we investigated potential differences between brain regions. Visual inspection revealed no clear topographic patterns of between-group differences (Fig. 2). For generating this figure, we kept only subjects with recordings at both 6 and 12 months (TLA: *N* = 54; ELA-noASD: *N* = 42; ELA-ASD: *N* = 16) and we averaged connectivity between the two time points. For comprehensiveness, we also included in supporting documents an additional statistical analysis investigating regions that could have been over or underconnected (see Additional file 1: Tables S3 and S4). No significant relationships were replicated between sites, although this might be due to low power.

**Fig. 3:**
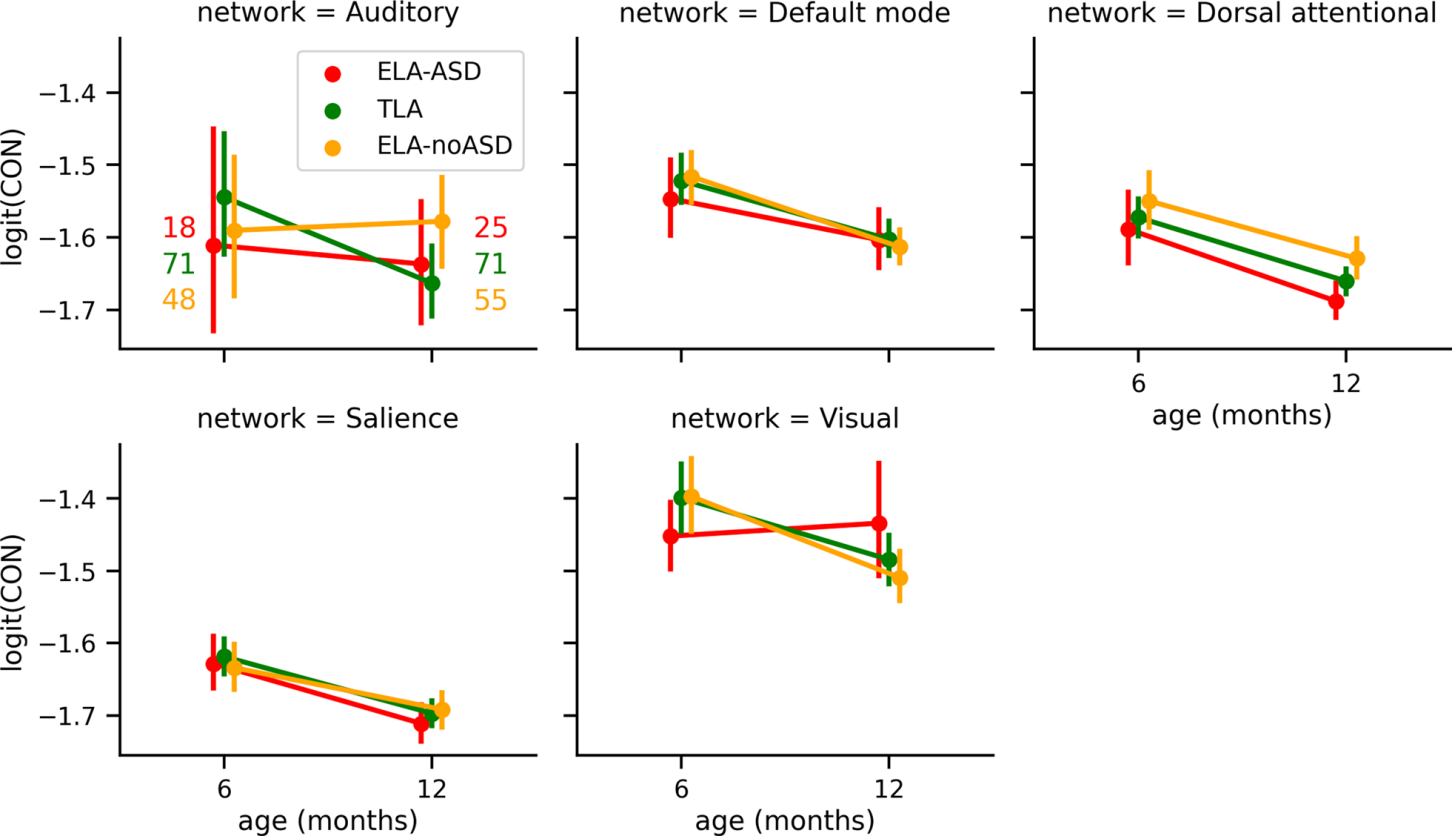
Logit-transformed CIPLV values within the different resting-state networks. Displayed per network (different panels), for the different time points (*x*-axis), and diagnostic groups (color). Whiskers represent the bootstrapped 95% confidence interval. Sample sizes indicated on the first panel are the same for all networks

Further, comparing average connectivity within resting-state networks did not support statistically significant group differences in connectivity between functional networks (Fig. 3).

**Fig. 4:**
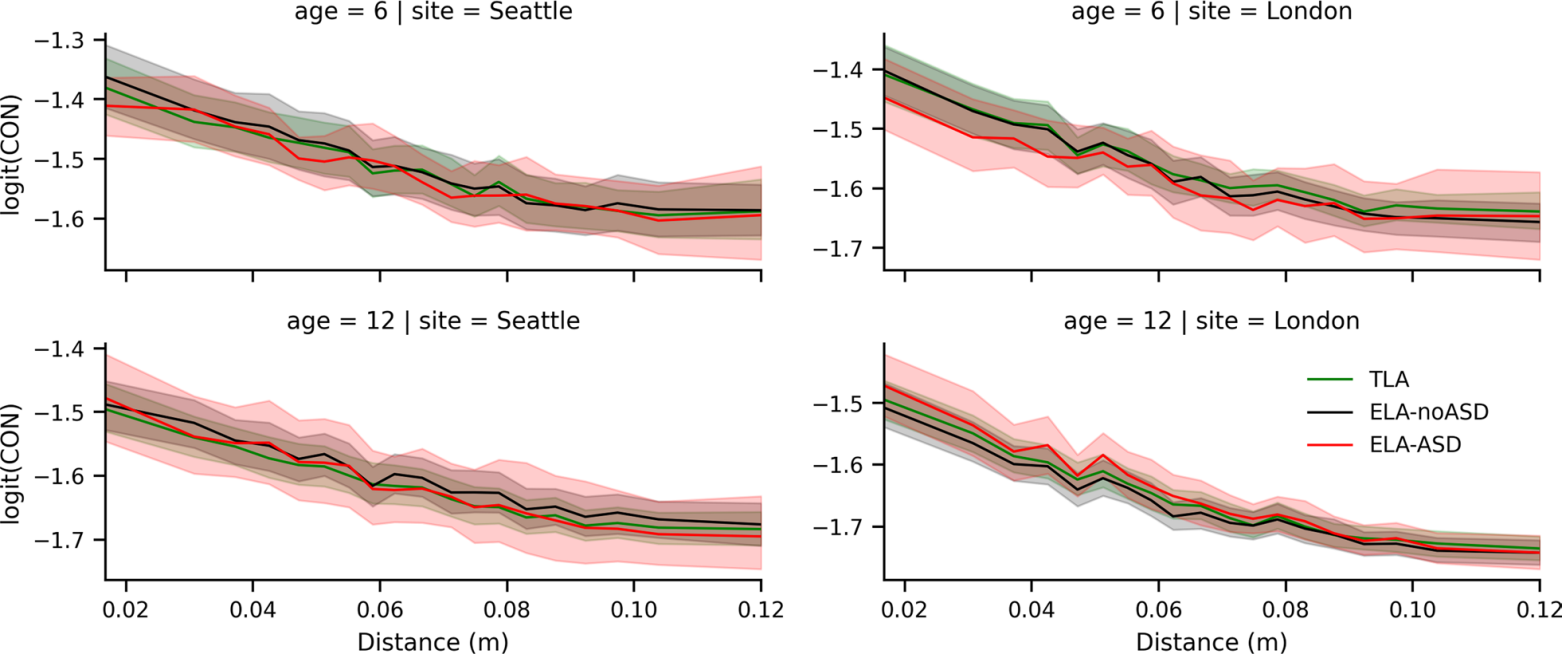
Average logit-transformed CIPLV connectivity as a function of the distance. Displayed between regions (*x*-axis), age (rows), site (columns), and group (color). To smooth these lines, distances are split into 20 bins, each covering 5% of the distribution. Shaded regions show 95% bootstrapped confidence intervals

Similarly, we verified if between-group differences were modulated by the distance between brain regions (Fig. 4) since there are reports of long-range underconnectivity and potentially short-range overconnectivity in ASD [12]. Again, we did not see any evidence for such a relationship. No formal statistical testing was performed for Figs. 3 and 4 since bootstrapped confidence intervals did not suggest the potential presence of significant group effects with current sample sizes.

### Exploratory analysis: dimensional association with ADOS CSS

**Fig. 5:**
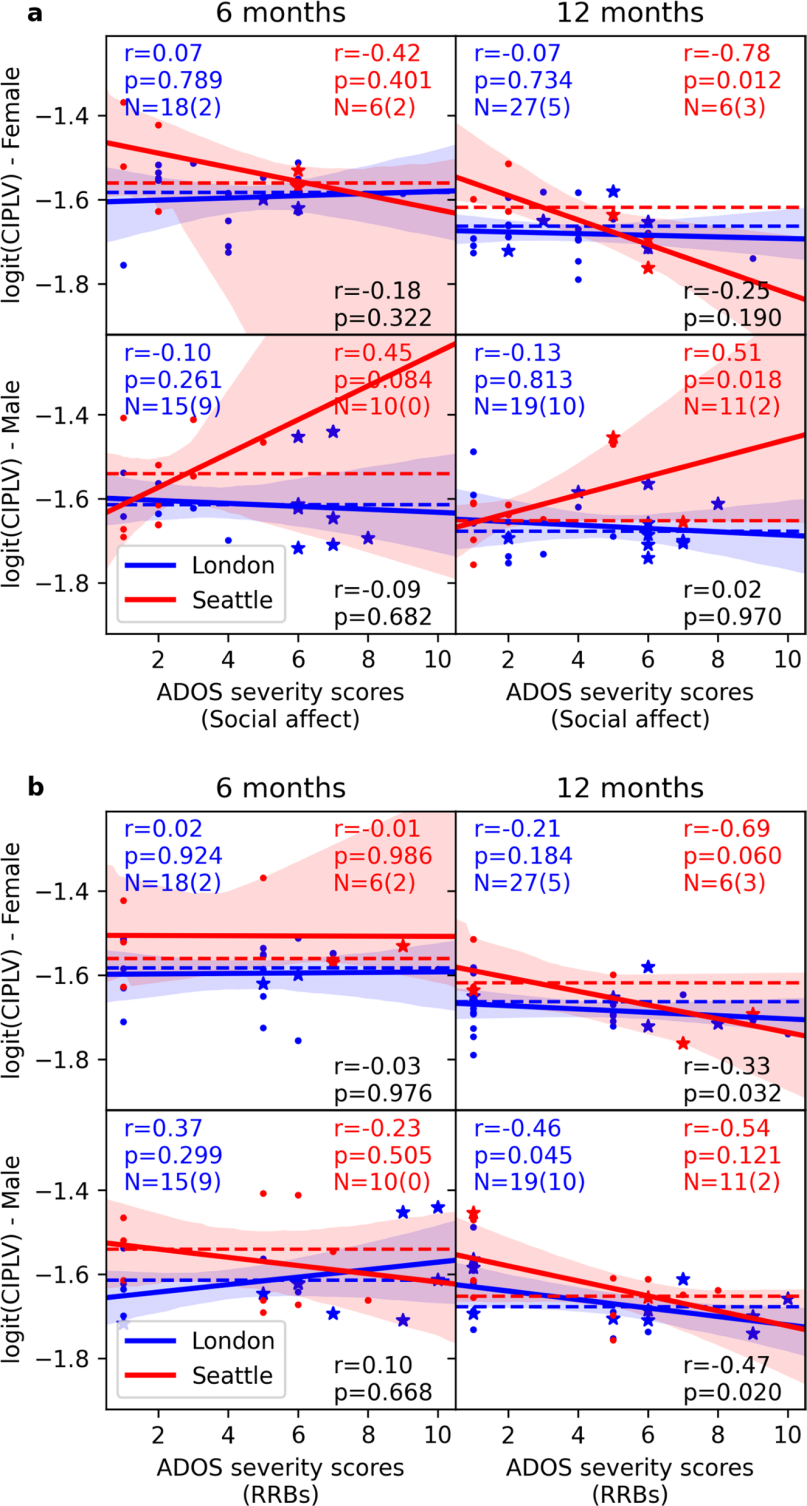
Regression between the logit-transformed CIPLV connectivity and ADOS calibrated severity scores for the ELA infants. Displayed per sex (rows), time point (columns), and sites (blue: London; red: Seattle; black: Pooled). The dashed lines indicate the average connectivity for TLA infants. Pearson’s coefficients of correlation (*r*) are indicated, along with *p* values from robust linear univariate regressions. Stars indicate participants diagnosed with ASD, whereas dots indicate individuals without ASD. Samples sizes are indicated for both sites (sample sizes for ELA-ASD are shown in parenthesis). Sample sizes for the pooled dataset have been omitted to save space but equal the sum of the sample sizes for both sites. **a** Social affect. **b** RRB. Similar plots for the overall ADOS CSS are shown in Supplementary Fig. 7

Considering that our exploratory analysis of group-level differences did not yield any striking differences, we performed a similar exploratory analysis to examined dimensional associations between functional connectivity and ADOS CSS within ELA participants (Fig. 5). With pooled datasets, we observed a statistically significant negative correlation between functional connectivity at 12 months and RRB for males (*p* = 0.020; Pearson’s *r* = − 0.47) and females (*p* = 0.032; Pearson’s *r* = − 0.33). Interestingly, for the Seattle dataset, at 12 months, the functional connectivity was negatively correlated with social affect for females (*p* = 0.012; Pearson’s *r* = − 0.78), whereas it was positively correlated for males (*p* = 0.018; Pearson’s *r* = 0.51). This result was, however, not replicated for the London dataset or for the pooled dataset.

It should be noted that many participants (52 in London; 36 in Seattle) have been assessed for ADOS CSS at two time points, typically around 24 and 36 months. Since there are some differences in these scores at different time points (in particular, the London site shows low correlations between time points; see Additional file 1: Figure S8), differences in ADOS CSS (e.g., due to lack of stability of ASD symptoms across the observation period leading particularly to false negative in early assessments [47]) could impact our results. The correlations we previously reported were based on the earliest ADOS CSS available. To validate the impact of using ADOS CSS obtained at different time points, we also replicated our analysis using the ADOS CSS from the oldest time point available. Using this alternative ADOS assessment, we also observed that the functional connectivity was negatively correlated with RRB, but only in males and at both 6 (*p* = 0.004; Pearson’s *r* = − 0.42) and 12 months (*p* = 0.029; Pearson’s *r* = − 0.41) (see Additional file 1: Figure S5). The correlations with social affect also tended to be of inverted magnitude in the Seattle dataset but reached statistical significance only for the males at 6 months (*p* = 0.013; Pearson’s *r* = 0.45).

Again, it is possible that our sample size does not allow us to detect all existing relationships. For reference, a sample size of 29 participants is necessary to detect at a statistical significance level of 0.05 and a power of 0.8 a large effect size (*r* = 0.5) for a Pearson’s coefficient of correlation. Although we used a robust regression (Huber regressor) to calculate statistical significance, this sample size provides a reasonable approximation. Also, it should be noted that reported *p* values are not corrected for multiple tests, and p-values for multiple correlations are reported in Fig. 5. Thus, these results should be interpreted with care and be considered as hypotheses to be corroborated in confirmatory analysis. In our analysis, we reported results for each site separately to look at replicability between sites, but the relatively small sample sizes did not support replicated statistically significant results across sites. Setting aside site replicability, pooled results for two ages, two sexes, and two dimensions (social and RRB) results in eight tests, and a corrected significance threshold at 0.05/8 according to the Bonferroni correction. No result passed this conservative threshold.

For comprehensiveness, we also tested for potential region-specific relationships. No regions resulted in a statistically significant effect that is reliable across sites, although a negative correlation between functional connectivity and RRB CSS was found for the caudal middle frontal region of the left hemisphere for both sites (London: *r* = − 0.337, *p* = 0.012; *n* = 45; Seattle: *r* = − 0.393, *p* = 0.036, *n* = 29) with a corrected *p* value marginally significant (p = 0.058) for the combined observation at both sites. We also note that most reliably high correlations (above 0.2 or under − 0.2 for both sites) were in frontal regions. Most of these were for RRB, and all these correlations were positive at 6 months (5 regions), and negative at 12 months (3 regions), aligning well with previous reports of initial overconnectivity followed by underconnectivity in ASD [12]. The details of this analysis are presented in supplementary documents (see Additional file 1: Table S5-S7).

## Discussion

In this study, we used the EEG-IP database to examine whether functional connectivity between EEG cortical sources during the first year of life is atypical in ELA infants later diagnosed with ASD. It constitutes one of only a handful of studies on EEG functional connectivity in infants with ASD [15, 16, 18, 19, 48, 49]. Furthermore, it uses methods that improve upon past studies, such as using a robust functional connectivity metric and computing connectivity over cortical sources using age-matched head templates.

### Underconnectivity in infants with ASD

Our observations provide some insights into the developmental origins of underconnectivity in children and adults with ASD. They do not clearly support an underconnectivity hypothesis in very early childhood during the pre-diagnostic period. However, correlations between ADOS and functional connectivity, when significant, tended to be of negative sign (i.e., higher ADOS were associated with less functional connectivity; see Fig. 5 and Additional file 1: Figures S7 and S9). Also, although not statistically significant, we note that most regions that had reliably at least a medium Cohen’s d effect sizes (> 0.3) across sites were indicating underconnectivity (9/10) and were for comparisons between ELA-ASD versus controls (8/10) (see Additional file 1: Tables S5 and S6). This observation contrasts with a similar dimensional analysis within the ELA group that suggests more positive correlations with social affect and RRB CSS scales at 6 months (8/8; Additional file 1: Tables S5 and S6) and negative correlation with the RRB CSS scales at 12 months (3/3; Additional file 1: Tables S7). Nevertheless, our results are inconclusive with respect to a relationship between EEG functional connectivity and autism in infants (Additional file 1: Tables S3 and S4). Potential effects may be too small to reliably detect with our sample size or too difficult to capture through population averages due to sample heterogeneity. We also note that such inconclusive results are compatible with recent reports from the large sample study LEAP [50].

Beyond generalized group differences in the all-to-all connectome, we also looked for different subsets of connections (e.g., belonging to specific functional networks) to investigate the possibility of a more specific neural effect. Our investigation failed to reveal a systematic, reliable, and reproducible pattern across the two sites. This situation may be due to a few factors. First, averages may not contrast groups if the effect of ASD on functional underconnectivity is inconsistent across subjects (e.g., a heterogeneous mixture of over and underconnectivity may end up showing a normal level of connectivity at the group level). Further, infant EEG is inherently noisy, and the experimenters have little control over the infant’s behavior due to uncontrollable factors such as tiredness and fussiness of the infants, which are likely to cause variable brain activations within and between subjects during EEG acquisitions. Lastly, we noted what looks like a significant degree of source leakage. Similar to volume conduction between scalp channels, source leakage generates zero-lag correlations between brain regions. It is hard to know what portion of unlagged synchrony is due to genuine zero-lag connectivity known to exist even between distant brain regions [51, 52] and what part is due to source leakage and unresolved challenges associated with the under-determined estimation of cortical sources from scalp signals. Regardless of its cause, such source leakage blurs regional specificity by increasing the apparent similarity of brain activity between regions.

### Effect of biological sex on the relationship between ASD and functional connectivity

In our analyses, we observed different connectivity profiles between elevated-likelihood females and males, with females (male) at 12 months showing a negative (positive) association between functional connectivity and social affect, as measured by ADOS CSS. This difference was visible in one of the two sites (Seattle) only, although the relatively small samples limit the possibility of detecting such effects reliably across sites, and the report of multiple tests exposes these unreplicated results to false positives. Thus, this result would need confirmation by future studies. Given the preponderance of autism in males, biological sex has emerged as a potential protective mechanism that mitigates the likelihood of developing ASD [53]. With respect to social affect symptoms, females appear to be more resilient, requiring higher genetic loading to reach the ASD diagnostic threshold [54]. Greater social cognitive abilities in females might contribute to such resilience and may be reflected in anatomical brain differences such as a comparatively thinner cortical sheet in several brain regions in females [55]. Our findings in infants could reflect early neurodevelopmental divergent biological sex trajectories that perhaps contribute to reduced prevalence in females with higher functional connectivity.

Lastly, we note that in this paper we follow the World Health Organization definitions for sex and gender, thus when discussing biological sex differences, we refer to differences currently thought to be influenced by biological and genetic properties. Still, we acknowledge that it is hard to completely disentangle the effects of biological sex and gender socialization in human development, particularly considering that gender socialization begins at birth and may influence neurobiology [56–58]. We would also like to acknowledge that autistic individuals may be less likely to identify with their sex assignment from birth compared to neurotypical individuals [59, 60], and while it is not possible to assess gender identity in infancy, we nonetheless encourage future autism studies to consider both gender and biological sex factors when possible.

### RRB CSS and functional connectivity

For the pooled dataset, the correlation between RRB CSS and functional connectivity was statistically significant for both males and females (female: *p* = 0.032; male: *p* = 0.020) at 12 months, but not 6 months. Although these p-values were not corrected for multiple testing, the fact that this result is significant for both sexes is noteworthy, and the probability of obtaining such p-values for both sexes (i.e., 0.032 × 0.020 = 0.00064) would survive a Bonferroni correction for 78 independent tests. Further, it supports previous results in the literature linking functional connectivity and RRB [22] for the London sample. That study found this relationship for the same age (i.e., around 14 months), using a different measure of functional connectivity (debiased weighted phase lag index) and looking specifically at alpha band activity (7–8 Hz). The authors associated this result with frontal-striatal circuits, although they did not perform source reconstruction. Our results were found for global connectivity, but when looking for localized functional connectivity, mostly frontal regions showed elevated correlations. Further refining these results would require a larger sample size to compensate for the loss of power associated with the correction for multiple tests.

### Limitations

This analysis was performed on a dataset pooled from two methodologically similar but independent studies. Of course, such a pooling introduces heterogeneity in the overall sample and methods (e.g., systematic between-site differences in ADOS administration), and it can cause issues due to a site effect and unbalanced subsamples. The main analysis addresses this issue by explicitly including a site regressor in both linear models (main effect and interactions). In the exploratory analyses, this effect is absent from results like those presented in Fig. 5 as we explicitly stratified by sites. Figures presenting the results per site allow a direct evaluation of between-site reproducibility. Further, previous publications reported extensive analyses to validate that our pre-processing pipeline was supporting a reliable pooling of data from different sites for this dataset [23, 24]. We nevertheless acknowledge that this study is a post hoc analysis of a pooled dataset and, as such, does not benefit from the advantages of a prospective analysis plan or study preregistration.

This study has been limited mostly due to issues related to small sample sizes inherent in sibling studies. In a previous systematic review, we have shown that studies on the impact of ASD on EEG and MEG functional connectivity often report contradictory results, probably due to many confounding factors across studies (e.g., differences in inclusion/exclusion criteria, in connectivity metrics, in frequency bands, in participant demographic characteristics) and methodological difficulties in estimating reliably the cerebral sources of EEG/MEG activity and the functional connectivity between them [12]. This review also showed that small sample sizes are often used in studies of functional connectivity in autism. Histograms of sample sizes used in these studies show that samples of 10 ASD subjects or less are not uncommon (24%), and most studies (74%) have ASD groups of no more than 25 subjects (see Additional file 1: Figure S6).

Our study compares relatively well, with an average size for the ASD group of 22 participants across time points (sites combined). This is particularly true considering the prospective nature of this study, i.e., although a comparatively large number of infants enter the study at young ages, only a fraction of them are later diagnosed with ASD. For this reason, the non-autistic groups are significantly larger than the sample of participants with ASD. Actually, our dataset constitutes the largest infant sample and the second-largest sample overall among the functional connectivity studies included in our previous review.

Nevertheless, our analyses have been limited by relatively small sample sizes. The first reason for that is that infant EEG is noisier than adult EEG because it is harder to control the sources of physiological artifacts (e.g., EMG and EOG contamination, movements, etc.) and the behavior or the attention of infants. Thus, a larger proportion of subjects end up discarded due to poor recording quality and kept data are generally more variable (noisy). The second reason is due to the unbalanced distribution of participants with or without ASD that results from the prospective nature of studies in ELA infants. This imbalance causes much smaller effective sample sizes than the number of tested subjects (see the section *Effect of group imbalance on statistical power* in Supporting Material). Thus, in summary, although this study involved a large number of participants, our analyses are still limited by our sample size. This conclusion again stresses the need for future studies to reach larger effective sample sizes to support decorticating the complex interactions between the many factors (e.g., age, biological sex, symptom severity, brain region, frequencies) that may confound our understanding of the relationship between functional connectivity and autism. Further, improving the diversity of these samples (e.g., by including other early predictors of ASD such as preterm birth or genetic conditions) may also be instrumental in mitigating the possibility of ELA defined by sibling studies being unrepresentative of other subgroups of autistic individuals.

## Conclusion

In summary, our analyses suggest relatively small and unspecific effects of elevated ASD likelihood on EEG functional connectivity, potentially due to heterogeneity in how functional connectivity abnormalities present themselves in different subjects. It nevertheless indicated potential sex-specific differences in how functional connectivity correlates with later social symptoms, whereas RRB seems to be associated with functional connectivity independently from biological sex. We obtained these results using a recently published connectivity measure (CIPLV) that solves many issues previously observed for similar measures. Further, we benefited from newly released structural head templates for infants to perform connectivity analysis between EEG sources rather than between EEG scalp signals, resulting in connectivity measures that can more easily be associated with brain regions and that are less likely to be confounded by known issues such as volume conduction and common sources. Our observations of ADOS CSS correlating with functional connectivity more reliably for RRB might indicate that connectivity could be useful in distinguishing different symptom profiles. Also, sex differences in how functional connectivity correlates with social affect might reflect sex-specific resilience to specific ASD symptoms. Nevertheless, since the results from the main analyses were mostly null findings and the more specific associations with ADOS CSS dimensions were obtained through exploratory analyses, these results need corroboration in future confirmatory studies.

### Supplementary Information

**Additional file 1**. The supporting information contains additional methods, additional results, Supplementary Figures 1–10, and Supplementary Tables 1–7.

## Data Availability

The datasets constituting EEG-IP and analyzed during the current study are available from the lead of the original studies, upon reasonable request to basis@bbk.ac.uk for the London dataset and to sjwebb@uw.edu for the Seattle dataset. The code used for the analysis is publicly available at https://github.com/lina-usc/asd_infants_eeg_con_paper.

## Abbreviations

ADOS: Autism diagnostic observation schedule
ASD: Autism spectrum disorder
CIPLV: Corrected imaginary phase-locking value
CSS: Calibrated severity scores
EEG: Electroencephalogram
EEG-IP: International infant EEG platform
ELA: Elevated likelihood for autism
EOG: Electrooculogram
fMRI: Functional magnetic resonance imaging
MEG: Magnetoencephalogram
PLI: Phase lag index
RRB: Restrictive and repetitive behaviors
TLA: Typical likelihood for autism
wPLI: Weighted PLI
