# Supplementary material for: EEG functional connectivity in infants at elevated familial likelihood for autism spectrum disorder
Source: Mol Autism. 2023 Oct 7;14:37. doi: 10.1186/s13229-023-00570-5 (PMC10559476; doi:10.1186/s13229-023-00570-5)
Supplement: Supplementary file 1 — Additional file 1. The supporting information contains additional methods, additional results, Supplementary Figures 1–10, and Supplementary Tables 1–7. [file 13229_2023_570_MOESM1_ESM.pdf]

Supporting information

Additional methods

Controlling for sample size bias in the estimation of functional connectivity

Since CIPLV is biased by the sample size (Supplementary Figure 1.a), we computed connectivity using the same number of epochs (N_{sel}) for each subject. However, this approach presents a challenge since there is a large difference in the number of available epochs (N_{tot}) between recordings. To avoid rejecting a significant amount of recordings because they do not have enough valid data, we have to use a relatively low value for N_{sel}: 20 1-s epochs.

Figure 1.b illustrates the tradeoff in choosing the optimal value for N_{sel}. We used bootstrapping to compute the functional connectivity as the mean value of a set of N estimates based on N_{sel}-epoch random subsamples (without replacement), with N taken as twice the value of N_{tot} divided by N_{sel}. For example, for a recording with N_{tot}=100 epochs, this rule results in N=2xN_{tot}/N_{sel}=2x100/20, or 10 estimates. This way, most epochs (but not all, since the selection is random) are used to obtain the bootstrapped estimates, with epochs being used on average twice. This approach ensured we took advantage of most of the available data while eliminating the bias due to differences in the number of available epochs across subjects, sites, and time points.

Reliability of CIPLV

To illustrate the superior reliability of CIPLV estimates compared to the often-used weighted PLI (wPLI) measure, we bootstrapped the estimation of the CIPLV and wPLI for 100 iterations and computed the mean and the standard deviation of these samples for every pair of channels. Figure 1.c-e shows the distribution of these standard deviations (c), mean (d), and the ratio

std/mean (e) across pairs of regions. Standard deviations are expected to grow larger with larger mean values, which is not indicative of lesser reliability but of the data being on a different scale. This difference in scaling can be canceled out by normalizing the standard deviations by the corresponding mean values. The larger distribution of the normalized standard deviations of wPLI indicates that this measure tends to be less reliable across samples than CIPLV. This illustrative example is based on a single randomly chosen subject but reflective of the increased reliability of CIPLV we observed consistently during our preliminary analyses.

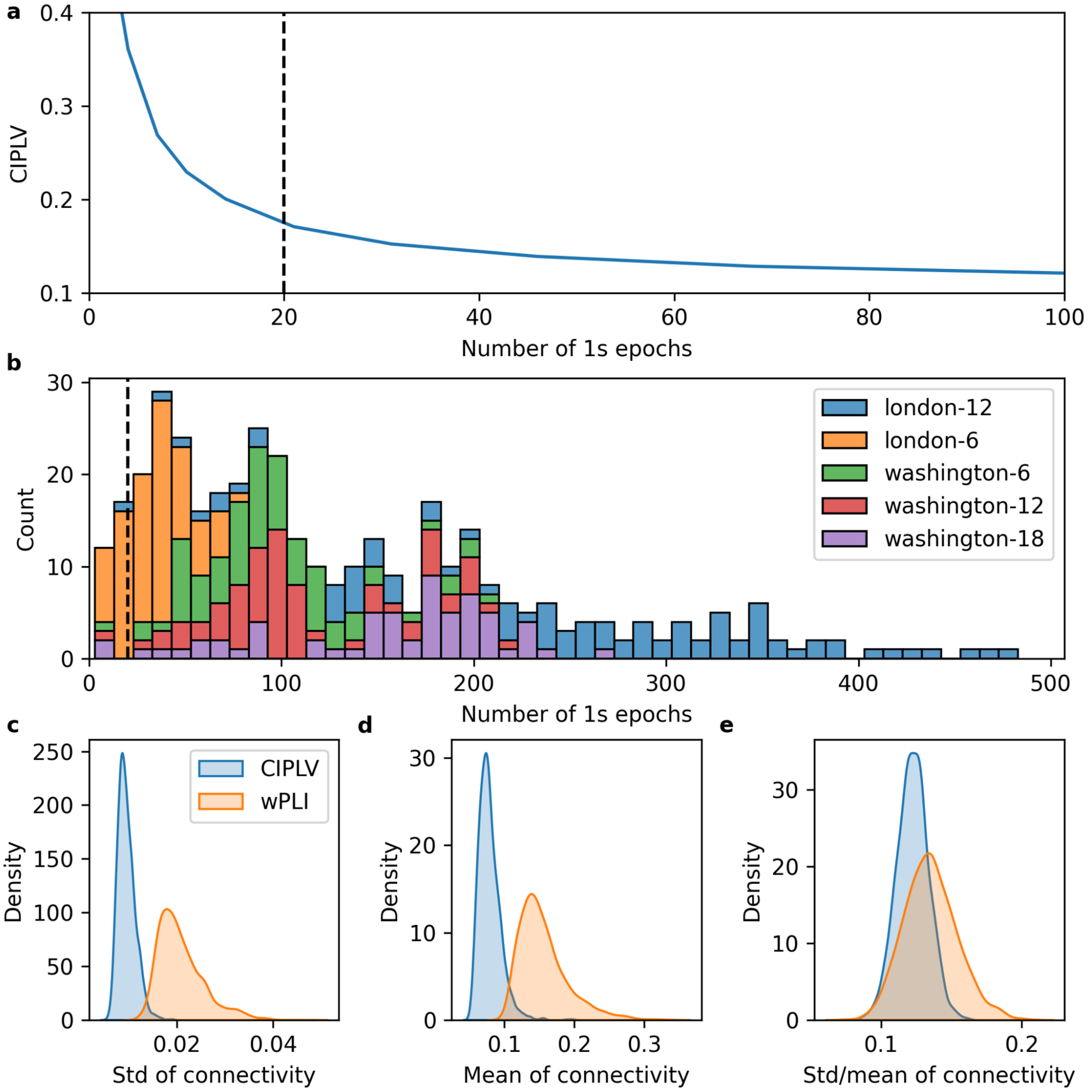

Supplementary Figure 1. a) Average CIPLV estimates when using subsets containing the numbers of epochs (*N_{sel}*) specified along the x-axis. b) Histogram of the number of clean epochs available for analysis across the different sites and time points. By using a threshold *N_{sel}* at 20 epochs (black dashed line), we reject 21 recordings on the grounds that they did not have enough epochs to allow for reliable estimation of functional connectivity. b-d) Distribution of the standard deviation (std) (b), mean (c), and std/mean (d) values for the CIPLV and wPLI measures for all pairs of regions for a randomly picked subject. Statistics (mean, std, std/mean) are computed across bootstrapped samples per pair of regions, whereas plots show the distribution of these statistics across region pairs.

Outlier rejection

We rejected the EEG recordings in which the functional connectivity was considered a statistical outlier (see Supplementary Figure 2) using the thresholds defined as follows

$$th_{min} = Q_1 - 1.5(Q_3 - Q_1) \quad (4.a)$$

$$th_{max} = Q_3 + 1.5(Q_3 - Q_1) \quad (4.b)$$

where Q1 and Q3 represent the first and third quartiles, respectively, and the 1.5 factor is such that for a normal distribution, these thresholds correspond to rejecting points more extreme than 99.3% of the distribution. We chose these non-parametric thresholds because they offer more stability against departure from normality or symmetry than would thresholds relying on parameters of the normal distribution such as the mean and the standard deviation. This procedure resulted in the rejection of 11 recordings, distributed as follows:

- month: 6: 4/141 (2.8%); 12: 7/158 (4.4%)

- group: TLA: 7/149 (4.7%); ELA-noASD: 3/106 (2.8%); ELA-ASD: 1/44 (2.3%)
- site: London: 5/157 (3.2%); Seattle: 6/142 (4.2%)
- sex: F: 8/146 (5.5%); M: 3/153 (2.0%)

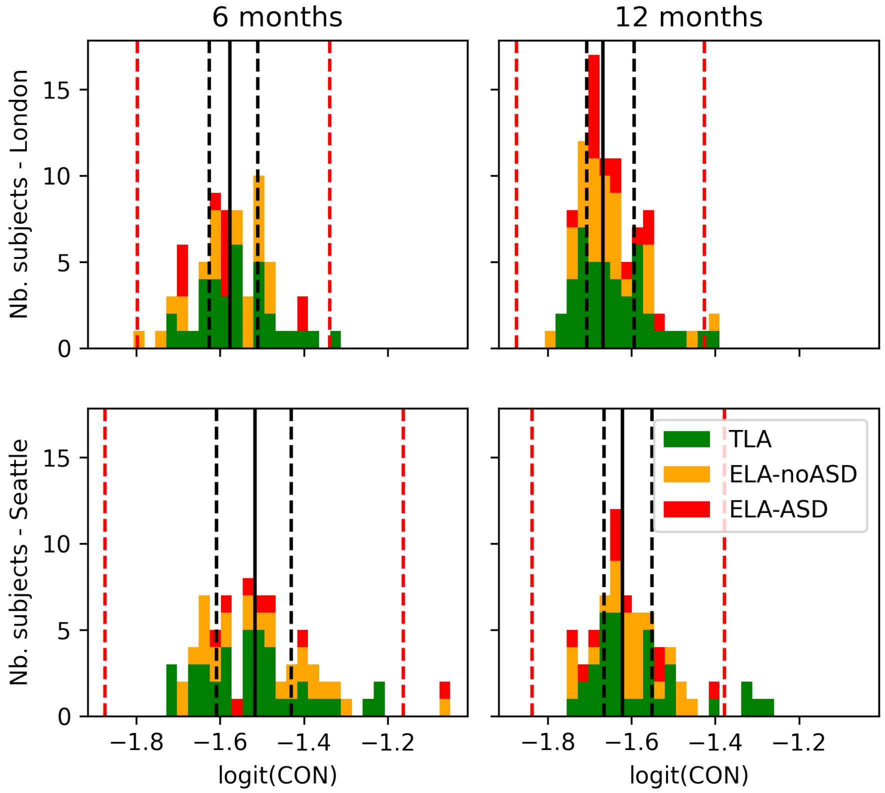

Supplementary Figure 2. Stacked histograms illustrating the rejection of statistical outliers. Vertical lines show the median (solid black lines), the first and third quartiles (dashed black lines), and the threshold defined in (4) (dashed red lines). Rows and columns separate the recording site and the age at the time of recording, respectively.

Rejection of channels and independent components

We interpolated channels flagged as containing artifacts using spherical splines, as implemented in MNE-Python. Similarly, we removed from the raw EEG all independent

components flagged as not representing neural signals. This procedure is described and validated in detail in [23]. Supplementary Figure 3 illustrates the distribution of dropped channels and independent components, split by site, sex, and group. We computed two mixed-effect linear regressions using the formula "Y ~ age + site + sexe + group", with Y taken as the percentage of bad channels and independent components, and with the participant identifier as grouping factor (i.e., random effect). The proportions of bad channels and components were impacted only by the site factor (channel: $p=7.7e-19$, component: $p=1.7e-21$; all other p-values > 0.05). Supplementary Figure 3 shows relatively large rejection proportions. This high level of rejection is partly due to the amount of noise present in infant EEG recordings. However, it is also due to a rather conservative inclusion of channels and components. This approach has been shown to be effective in avoiding rejecting more recordings than necessary and increasing the signal-to-noise ratio in EEG data, as illustrated by larger evoke-related potentials than when using alternative pipelines [23].

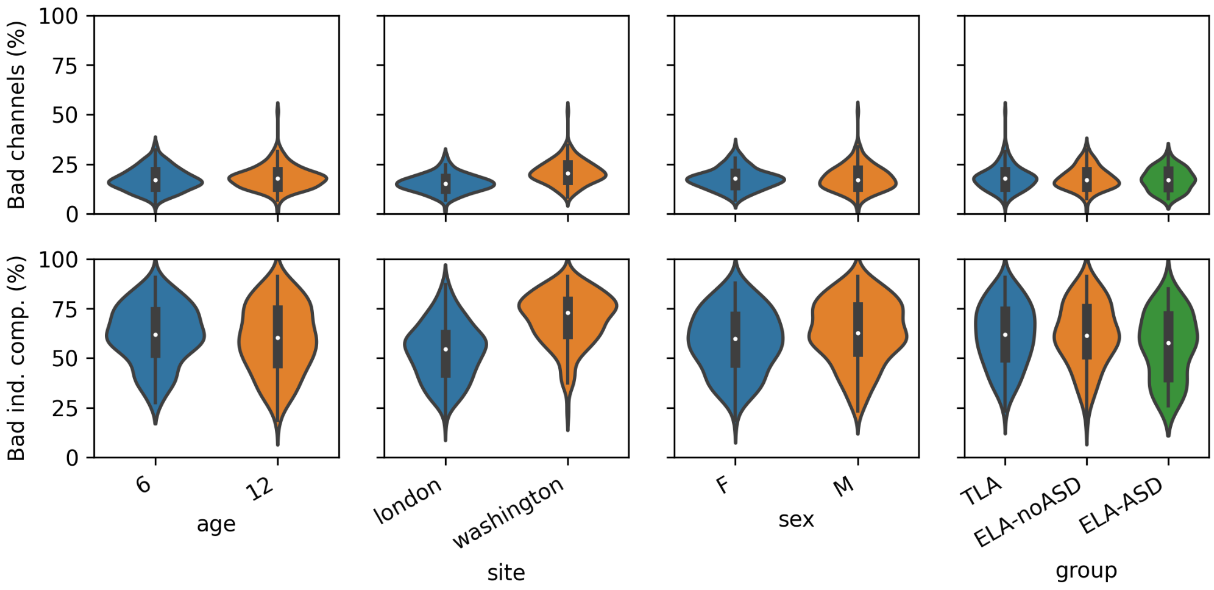

Supplementary Figure 3. Comparison of the percentage of bad channels and independent components rejected.

Effect of group imbalance on statistical power

Our analyses have been limited by decreased statistical power due to diagnostic group imbalance. To exemplify the effect of an imbalance between groups, we consider a fictive sample of 20 ASD subjects and 200 controls, and we suppose that we are interested in a measure that has a normal distribution with mean values of μ_1 and μ_2 for these two groups and the same standard deviation across groups σ=1. The standard error for these two distributions will be equal to $\frac{\sigma}{\sqrt{20}} = \frac{1}{\sqrt{20}} = 0.224$ and $\frac{\sigma}{\sqrt{200}} = \frac{1}{\sqrt{200}} = 0.071$, respectively. Consequently, the difference of the means will be a normal distribution with mean values $\mu=\mu_1-\mu_2$ and a pooled standard error equal to $se = \sqrt{\frac{1}{20} + \frac{1}{200}} = 0.235$. This standard error is the same as we would obtain with equal samples of 36.4 subjects. That means that our total unbalanced sample of 220 subjects has the same statistical power as a balanced sample of about 72 subjects (i.e., the effective sample size for this study is equal to 72 subjects, not 220). Thus, in our study, we do not benefit much from our larger sample of control subjects for group comparisons with ELA-ASD. We might further note that a longer follow-up might have increased the size of the ASD group, particularly for the Seattle site, since ASD often goes undetected at young ages (e.g., at 2 years old) [61]. This limitation is not present for comparison between ELA and TLA since these groups are balanced. The original studies were powered to study such group comparisons.

Additional results

Additional linear regression

The results of models (2) and (3) without averaging connectivity measures within recordings are shown in Supplementary Tables 1 and 2. These models show more significant p-values and

even some significant interactions with group and overall ADOS CSS. However, we note the absence of significance for the main effects of group and ADOS, which casts some doubts on the reliability of these interactions. Further, we prefer the more conservative model presented in the main text because of the difficulty of fully capturing the correlation structure between observations in models with large amounts of repeated observations. We consider this complexity to be more likely to lead to misleading results. For example, in this specific case, the connectivity measures establish a mapping between two sets of regions. Thus, these measures are likely to have a complex correlational structure, which would be hard to properly model without increasing significantly the complexity of these models, the number of parameters, and the difficulty of finding reliable parameter estimates. Thus, we believe the model using averaged connectivity to be more conservative and reliable, although it is likely to have less statistical power and more susceptible to false negatives.

Supplementary Table 1. Model (2), using all connectivity measures (no within-recording averaging).

Model:	MixedLM	Dependent Variable:	log_con
No. Observations:	599040	Method:	REML
No. Groups:	176	Scale:	0.0756
Min. group size:	2080	Log-Likelihood:	-77183.1460
Max. group size:	4160	Converged:	No
Mean group size:	3403.6		

	Coef.	Std.Err.	z	P>|z|	[0.025	0.975]
Intercept	-1.572	0.029	-54.608	0.000	-1.629	-1.516
group[T.ELA-noASD]	0.041	0.032	1.283	0.200	-0.021	0.102
group[T.TLA]	0.052	0.031	1.677	0.094	-0.009	0.112
sex[T.M]	0.031	0.032	0.980	0.327	-0.031	0.094
site[T.Seattle]	0.066	0.033	2.006	0.045	0.002	0.130
group[T.ELA-noASD]:sex[T.M]	-0.047	0.037	-1.281	0.200	-0.120	0.025
group[T.TLA]:sex[T.M]	-0.046	0.034	-1.345	0.179	-0.112	0.021
group[T.ELA-noASD]:site[T.Seattle]	0.012	0.037	0.330	0.742	-0.061	0.085
group[T.TLA]:site[T.Seattle]	-0.008	0.034	-0.235	0.814	-0.075	0.059
sex[T.M]:site[T.Seattle]	0.005	0.024	0.194	0.846	-0.042	0.051
age	-0.009	0.000	-22.941	0.000	-0.010	-0.008
group[T.ELA-noASD]:age	-0.003	0.000	-6.824	0.000	-0.004	-0.002
group[T.TLA]:age	-0.002	0.000	-5.198	0.000	-0.003	-0.001
sex[T.M]:age	-0.000	0.000	-0.276	0.782	-0.001	0.000
site[T.Seattle]:age	-0.001	0.000	-4.380	0.000	-0.002	-0.001
subject_no Var	0.005	0.003				

Supplementary Table 2. Model (3), using all connectivity measures (no within-recording averaging).

Model:	MixedLM	Dependent Variable:	log_con
No. Observations:	232960	Method:	REML
No. Groups:	70	Scale:	0.0712
Min. group size:	2080	Log-Likelihood:	-23036.4207
Max. group size:	4160	Converged:	Yes
Mean group size:	3328.0		

	Coef.	Std.Err.	z	P>|z|	[0.025	0.975]
Intercept	-1.562	0.024	-65.483	0.000	-1.609	-1.516
sex[T.M]	-0.093	0.034	-2.761	0.006	-0.159	-0.027
site[T.Seattle]	0.170	0.041	4.112	0.000	0.089	0.251
sex[T.M]:site[T.Seattle]	-0.013	0.040	-0.317	0.751	-0.090	0.065
adoscss_earliest	0.008	0.006	1.359	0.174	-0.004	0.020
adoscss_earliest:sex[T.M]	0.007	0.007	1.005	0.315	-0.007	0.022
adoscss_earliest:site[T.Seattle]	-0.005	0.009	-0.539	0.590	-0.023	0.013
age	-0.009	0.001	-16.729	0.000	-0.010	-0.008
sex[T.M]:age	0.008	0.000	18.680	0.000	0.007	0.009
site[T.Seattle]:age	-0.009	0.001	-17.720	0.000	-0.010	-0.008
adoscss_earliest:age	-0.001	0.000	-10.875	0.000	-0.001	-0.001
subject_no Var	0.005	0.004				

Effect of frequencies

Our preliminary analysis did not reveal reliable indications for analyzing the effect of frequencies. Further, systematically analyzing effects across frequencies would have presented issues related to multiple tests, which compounded with limited sample sizes and statistical power, was not viable. Nevertheless, for the sake of comprehensiveness, we provide in Supplementary Figure 4-6 a visualization of average connectivity per site, sex, age, group, and frequency.

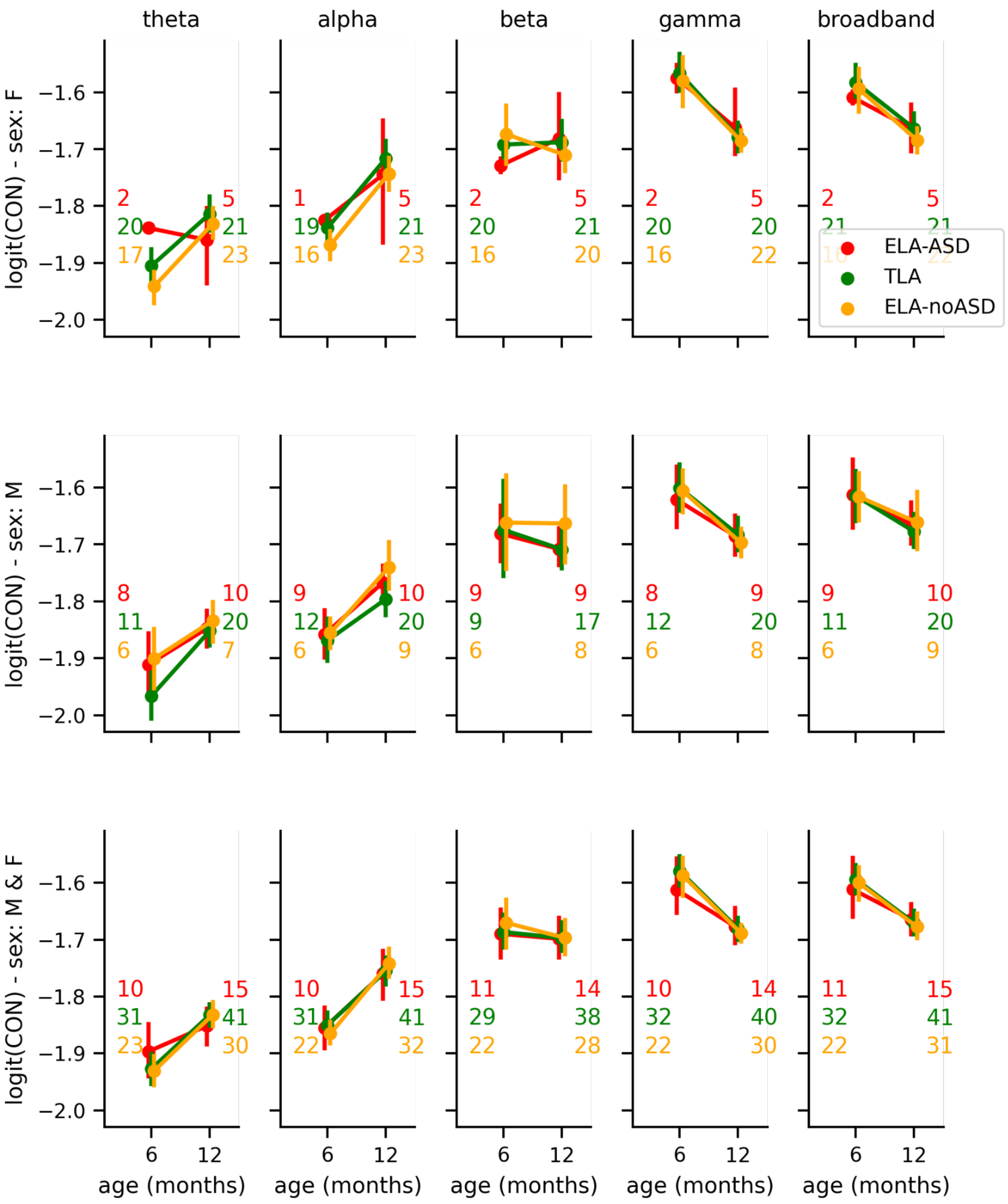

Supplementary Figure 4. Average connectivity across frequencies for the London site.

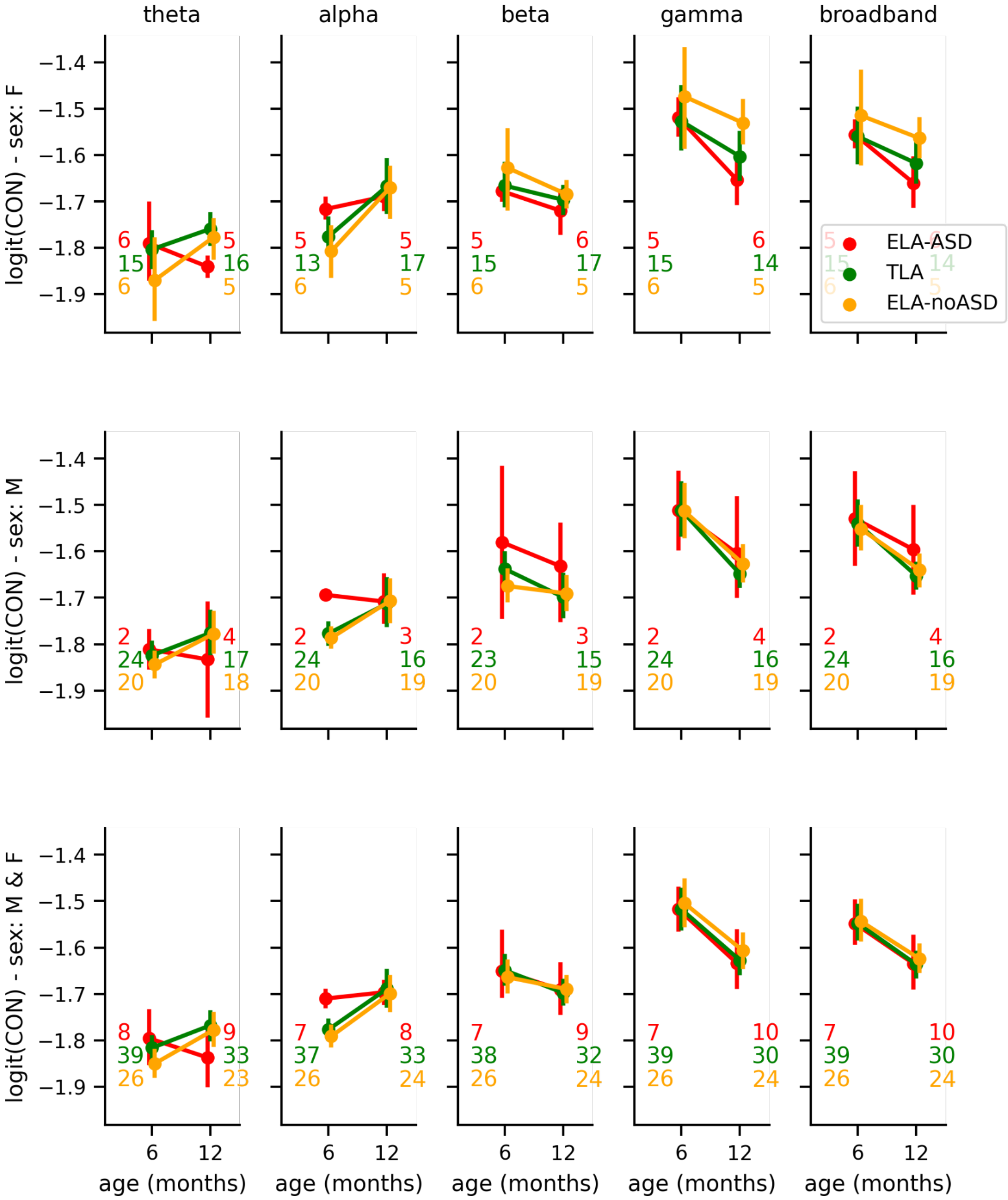

Supplementary Figure 5. Average connectivity across frequencies for the Seattle site.

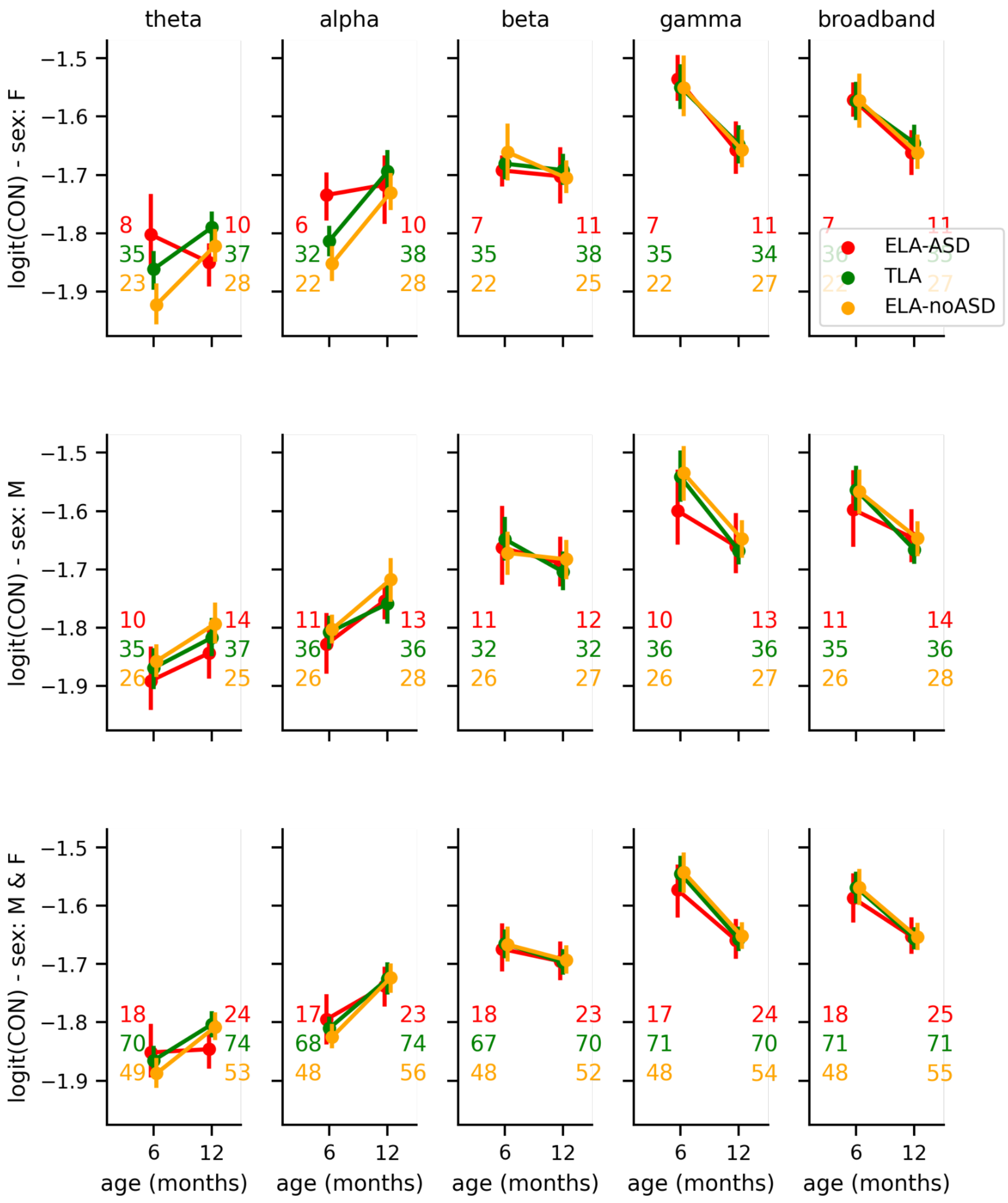

Supplementary Figure 6. Average connectivity across frequencies for the pooled dataset.

Additional statistical analysis of localized under and overconnectivity

To further document the visual analysis presented in Figure 2, we ran a statistical analysis to test for systematically over or underconnected regions. We first computed the average connectivity per region (i.e., we averaged the connectivity that a given region has with all other regions). To look for regions reliably over or underconnected across sites, we listed the regions that were showing Cohen's d statistics above 0.3 (Supplementary Table 3) or under 0.3 (Supplementary Table 4) at both sites. For these connections, we also reported the t-statistics and p-values from Student's t-tests for two independent samples. Although some p-values are below 0.05, none are consistently significant across both sites. Also, none of these p-values are sufficiently low to survive correction for multiple tests.

Supplementary Table 3. Connections with Cohen's d statistics above 0.3 for both sites.

region	age	contrast	London						Seattle					
			d	n1	n2	power	p	t	d	n1	n2	power	p	t
inferiorparietal-rh	12	ELA-ASD - TLA	0.578	15	41	0.469	0.061	1.916	0.519	10	30	0.283	0.164	1.421

Supplementary Table 4. Connections with Cohen's d statistics below -0.3 for both sites.

region	age	contrast	London						Seattle					
			d	n1	n2	power	p	t	d	n1	n2	power	p	t
lateraloccipital-lh	6	ELA-ASD - TLA	-0.456	11	32	0.247	0.199	-1.306	-0.492	7	39	0.216	0.238	-1.198
pericalcarine-lh	6	ELA-ASD - TLA	-0.732	11	32	0.534	0.043	-2.094	-0.430	7	39	0.176	0.301	-1.047
pericalcarine-rh	12	ELA-noASD - TLA	-0.472	31	41	0.498	0.051	-1.983	-0.426	24	30	0.332	0.126	-1.554
caudalanteriorcingulate-lh	6	ELA-ASD - TLA	-0.915	11	32	0.725	0.012	-2.618	-0.374	7	39	0.145	0.368	-0.910
caudalmiddlefrontal-lh	12	ELA-ASD - TLA	-0.367	15	41	0.223	0.229	-1.218	-0.687	10	30	0.450	0.068	-1.882
transversetemporal-rh	6	ELA-ASD - TLA	-0.322	11	32	0.147	0.362	-0.922	-0.558	7	39	0.265	0.181	-1.361
superiorparietal-rh	6	ELA-ASD - TLA	-0.417	11	32	0.214	0.240	-1.192	-0.319	7	39	0.118	0.441	-0.777
fusiform-rh	6	ELA-ASD - TLA	-0.315	11	32	0.142	0.373	-0.901	-0.350	7	39	0.133	0.399	-0.852
bankssts-rh	6	ELA-ASD - TLA	-0.312	11	32	0.141	0.377	-0.893	-0.512	7	39	0.230	0.219	-1.246

Additional statistical analysis of localized correlations between CSS scales and functional connectivity

We conducted an analysis similar to the previous one to evaluate statistically whether the relationship between ADOS CSS and functional connectivity depends on brain regions. Here again, we average connectivity per brain region and look for consistently large correlations (r > 0.2 or r < -0.2) across sites. Supplementary Tables 5-7 list the regions fulfilling this criterion for both the RRBs and social CSS scales. No region had r < -0.2 at both sites for the social CSS scale. P-values corrected for multiple testing have been computed as the multiplication of the p-values from both sites (i.e., the probability of both events happening at the same time) multiplied by the number of joint tests (i.e., Bonferonni correction for two age points X 65 brain regions). This correction for multiple tests is rather conservative (i.e., because of correlation between age points and brain regions, the equivalent number of independent tests is likely significantly smaller than 2 X 65), thus, although only one region was marginally statistically significant (p=0.058), this result is worth nothing. Also, the pattern of these results is interesting, with only positive correlations at 6 months and negative correlations at 12 months, mostly in frontal regions.

Supplementary Table 5. Regions with a Pearson's coefficient of correlation between the social CSS scale and average functional connectivity above 0.2 for both sites.

			London			Seattle			
	region	age	n	p	r	n	p	r	corrected_p
0	parsorbitalis-lh	6	32	0.300	0.201	31	0.028	0.371	1.085
1	middletemporal-lh	6	32	0.094	0.323	31	0.133	0.201	1.627
2	lateralorbitofrontal-lh	6	32	0.071	0.311	31	0.024	0.278	0.218

Supplementary Table 6. Regions with a Pearson's coefficient of correlation between the RRB CSS scale and average functional connectivity above 0.2 for both sites.

			London			Seattle			
	region	age	n	p	r	n	p	r	corrected_p
0	parsorbitalis-lh	6	32	0.193	0.264	31	0.443	0.223	11.129
1	medialorbitofrontal-lh	6	32	0.220	0.232	31	0.045	0.306	1.285
2	lateralorbitofrontal-lh	6	32	0.131	0.294	31	0.098	0.255	1.669
3	caudalmiddlefrontal-lh	6	32	0.104	0.358	31	0.134	0.258	1.814
4	supramarginal-lh	6	32	0.082	0.329	31	0.234	0.301	2.483

Supplementary Table 7. Regions with a Pearson's coefficient of correlation between the RRB CSS scale and average functional connectivity below -0.2 for both sites.

			London			Seattle			
	region	age	n	p	r	n	p	r	corrected_p
0	caudalmiddlefrontal-lh	12	45	0.012	-0.337	29	0.036	-0.393	0.058
1	lateralorbitofrontal-rh	12	45	0.021	-0.391	29	0.119	-0.268	0.329
2	parsorbitalis-lh	12	45	0.147	-0.236	29	0.184	-0.242	3.516

Additional Supplementary Figures

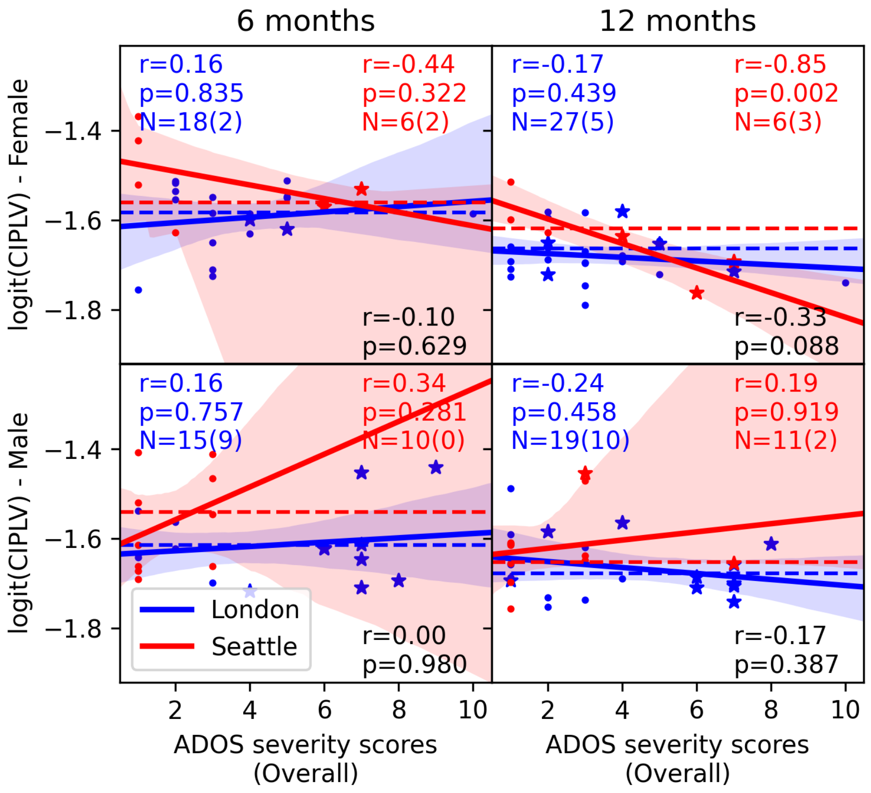

Supplementary Figure 7. Regression between the logit-transformed CIPLV connectivity and overall ADOS calibrated severity scores for the ELA infants, per sex (rows), time point (columns), and sites (blue: London; red: Seattle; black: Pooled). The dashed lines indicate the average connectivity for TLA infants. Pearson's coefficients of correlation (r) are indicated along with p-values (p) from robust linear regressions. Stars indicate participants diagnosed with ASD, whereas dots indicate neurotypical individuals.

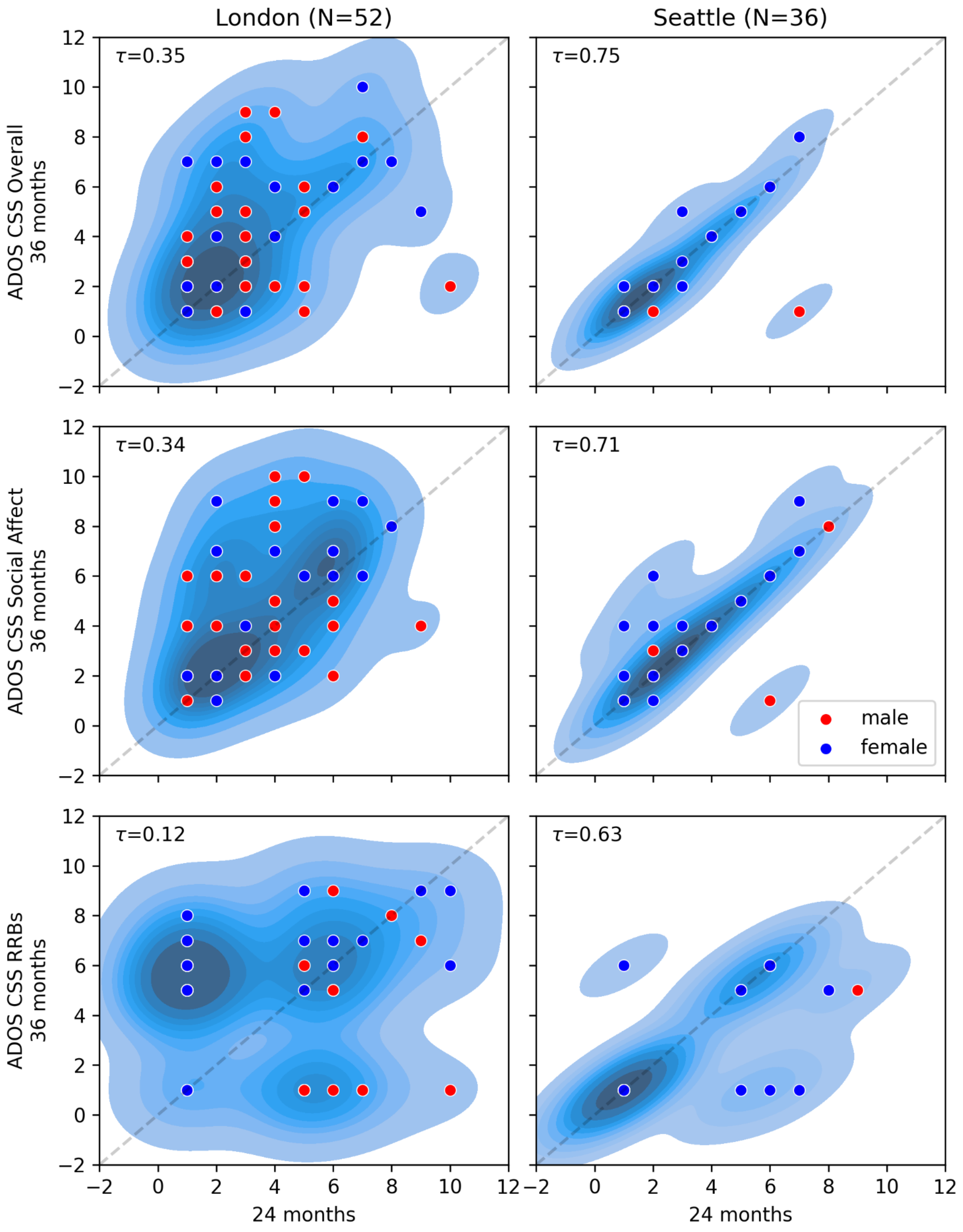

Supplementary Figure 8. Comparisons of ADOS scores collected first (around 24 months) and last (around 36 months) for subjects with two ADOS assessments. Kendall's tau non-parametric coefficient of correlation is shown in the upper left corner of each panel.

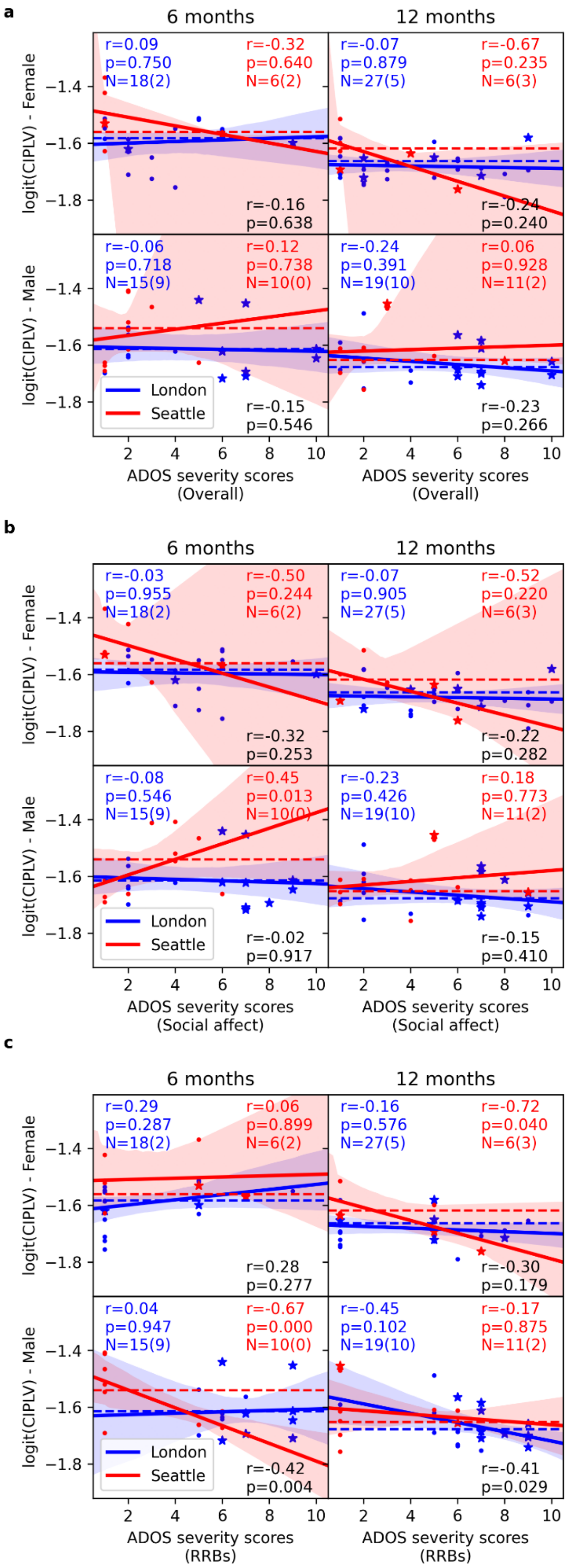
a
b
c
6 months
12 months
logit(CIPLV) - Female
logit(CIPLV) - Male
London
Seattle
ADOS severity scores
(Overall)
(Social affect)
(RRBs)
r=0.09
p=0.750
N=18(2)
r=-0.32
p=0.640
N=6(2)
r=-0.16
p=0.638
r=-0.07
p=0.879
N=27(5)
r=-0.67
p=0.235
N=6(3)
r=-0.24
p=0.240
r=-0.06
p=0.718
N=15(9)
r=0.12
p=0.738
N=10(0)
r=-0.15
p=0.546
r=-0.24
p=0.391
N=19(10)
r=0.06
p=0.928
N=11(2)
r=-0.23
p=0.266
r=-0.03
p=0.955
N=18(2)
r=-0.50
p=0.244
N=6(2)
r=-0.32
p=0.253
r=-0.07
p=0.905
N=27(5)
r=-0.52
p=0.220
N=6(3)
r=-0.22
p=0.282
r=-0.08
p=0.546
N=15(9)
r=0.45
p=0.013
N=10(0)
r=-0.02
p=0.917
r=-0.23
p=0.426
N=19(10)
r=0.18
p=0.773
N=11(2)
r=-0.15
p=0.410
r=0.29
p=0.287
N=18(2)
r=0.06
p=0.899
N=6(2)
r=0.28
p=0.277
r=-0.16
p=0.576
N=27(5)
r=-0.72
p=0.040
N=6(3)
r=-0.30
p=0.179
r=0.04
p=0.947
N=15(9)
r=-0.67
p=0.000
N=10(0)
r=-0.42
p=0.004
r=-0.45
p=0.102
N=19(10)
r=-0.17
p=0.875
N=11(2)
r=-0.41
p=0.029

Supplementary Figure 9. Same as Figure 5 and Supplementary Figure 7, but using the latest ADOS scores instead of the earliest. a) Overall. b) Social affect. c) RRBs.

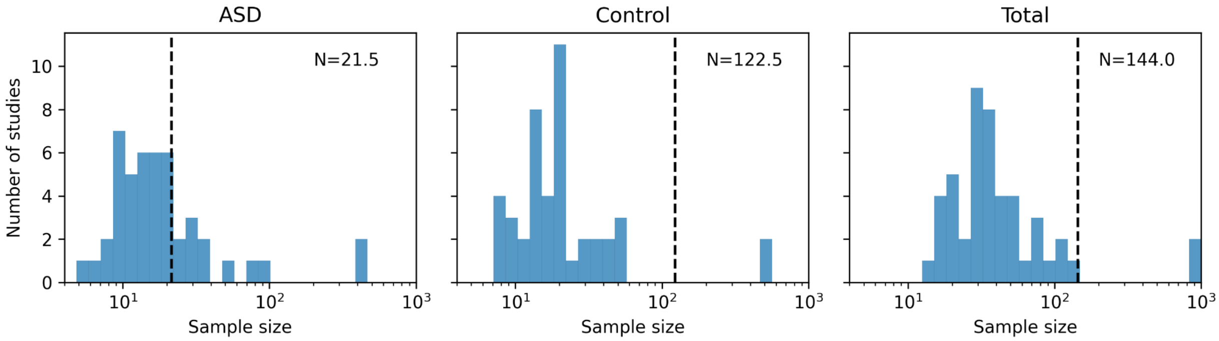

Supplementary Figure 10. Distribution of sample sizes across studies of EEG/MEG functional connectivity in autism, for the ASD (left), control (middle), and total (right) samples. Vertical dashed black lines indicate our sample size (average number of valid recordings for the two time points), pooled across sites.

Additional References

61. Zwaigenbaum, L., Bryson, S. E., Brian, J., Smith, I. M., Roberts, W., Szatmari, P., Roncadin, C., Garon, N., & Vaillancourt, T. Stability of diagnostic assessment for autism spectrum disorder between 18 and 36 months in a high-risk cohort. Aut Res. 2016;*9*:790–800.
